# Neurocognitive impairment and patient-proxy agreement on health-related quality of life evaluations in recurrent high-grade glioma patients

**DOI:** 10.1007/s11136-025-03984-1

**Published:** 2025-06-05

**Authors:** Ivan Caramanna, Martin Klein, Martin van den Bent, Ahmed Idbaih, Martin J. B. Taphoorn, Linda Dirven, Thierry Gorlia, Jaap C. Reijneveld, the EORTC Quality of Life Group

**Affiliations:** 1 Department of Medical Psychology, Amsterdam UMC, Vrije Universiteit Amsterdam, de Boelelaan 1118, PK 1Y 176, 1081 HZ Amsterdam, The Netherlands; 2 Brain Tumor Center Amsterdam, Amsterdam UMC, Vrije Universiteit Amsterdam, Amsterdam, The Netherlands; 3 Brain Tumor Center at Erasmus MC Cancer Institute, Rotterdam, The Netherlands; 4 Sorbonne Université, Inserm, CNRS, UMR S 1127, Institut du Cerveau Et de La Moelle Épinière, ICM, AP-HP, Hôpitaux Universitaires La Pitié Salpêtrière - Charles Foix, Service de Neurologie 2-Mazarin, 75013 Paris, France; 5 Department of Neurology, Leiden University Medical Centre, Leiden, The Netherlands; 6 Haaglanden Medical Centre, The Hague, The Netherlands; 7 European Organization for Research and Treatment of Cancer, Brussels, Belgium; 8 Department of Neurology, Amsterdam UMC, Vrije Universiteit Amsterdam, Amsterdam, The Netherlands; 9 Department of Neurology, Stichting Epilepsie Instellingen Nederland (SEIN), Heemstede, The Netherlands

**Keywords:** Quality of life, Glioma, Patient-proxy agreement, Neurocognitive functioning, PROs

## Abstract

**Purpose:**

The rate of missing data on patient-reported health-related quality of life (HRQOL) in brain tumor clinical trials is particularly high over time. One solution to this issue is the use of proxy (i.e. partner, relative, informal caregiver) ratings in lieu of patient-reported outcomes (PROs). In this study, we investigated patient-proxy agreement on HRQOL outcomes in high-grade glioma (HGG) patients.

**Methods:**

Generic and disease-specific HRQOL was assessed using the EORTC QLQ-C30 and QLQ-BN20 in a sample of 500 patient-proxy dyads participating in EORTC trials 26101 and 26091. Patients were classified as impaired or intact based on their neurocognitive performance. The level of patient-proxy agreement was measured using Lin’s concordance correlation coefficient (CCC), and the Bland–Altman limit of agreement. The Wilcoxon signed-rank test was used to evaluate differences between patients’ and proxies’ HRQOL.

**Results:**

Patient-proxy agreement in all HGG patients (N = 500) ranged from 0.399 to 0.743. Only 18.8% of all patients were neurocognitively intact. Lin’s CCC ranged from 0.231 to 0.811 in cognitively impaired patients and their proxies, and from 0.376 to 0.732 in cognitively intact patients and their proxies.

**Conclusions:**

The results of this study suggest that the moderate level of patient-proxy agreement observed in HGG patients would allow reliance on proxies' reports. However, the differences observed between neurocognitively impaired and intact patients stress the importance of taking into consideration patient’s clinical and neurocognitive status as well as their mental capacity for adequate clinical decision making in general and for PRO-related issues.

**Supplementary Information:**

The online version contains supplementary material available at 10.1007/s11136-025-03984-1.

## Introduction

The prognosis for high-grade glioma patients is quite dire, with a median survival time of 15 months and a usually rapid decline in general health. Therefore, it is not surprising that health-related quality of life (HRQOL) has become an important secondary outcome measure in high-grade glioma clinical trials, as a measure of patients’ functioning [1]. Traditional clinical trial outcomes such as progression-free survival or overall survival do not provide a complete picture of the patient’s functioning and well-being. Therefore, outcomes such as HRQOL and neurocognitive functioning are now typically included in brain tumor clinical trials, to better capture functioning and well-being. Unfortunately, many brain tumor clinical trials still suffer from a substantial amount of missing HRQOL data over time. [2] Restricting analyses only to patients able to offer complete patient-reported outcomes (PROs) might introduce a bias in clinical trials, since important HRQOL evaluations of patients with poor neurological and/or neurocognitive functioning might be missing [3]. A possible solution to this problem has been proposed in resorting to evaluations offered by partners, relatives, or informal caregivers, collectively referred to as “proxies” as substitute data in the analysis of missing scores reported by patients.

However, it is unclear to what extent proxy-reported outcomes are representative of the patients’ self-perceived outcomes. Previous studies in low-grade glioma patients showed that the level of neurocognitive functioning determines the degree of patient-proxy agreement. Moderate to high patient-proxy agreement was found in neurocognitively intact patients, while in patients with neurocognitive impairment, patient and proxy ratings differed regarding emotional functioning [4–7]. In general, there seems to be agreement between patients and proxies concerning physical functioning and symptoms, [6] but the same cannot be said regarding less visible issues such as mood and emotional functioning [8].

Furthermore, the debate concerning the subjectivity of HRQOL is still open. While HRQOL ratings are by definition subjective and should in principle be reported by the patient him- or herself, [9] High-grade Glioma (HGG) patients often face neurological and neurocognitive deterioration that could force clinicians to resort to proxy ratings because of the inability of the patient to do so and ensuring that patients who cannot respond for themselves are not excluded.

Questions concerning the ethical aspects of relying on proxy reports for HRQOL assessment arise, especially in the context of patients with neurocognitive impairment. The consensus is not to rely on proxy HRQOL estimates alone when possible but to use them in conjunction with patients’ self-reports [10, 11]. Furthermore, proxy assessments can also inform clinical decisions. In real-world clinical settings, incomplete patient HRQOL information can hinder effective relief of suffering and suboptimal decision-making. Patients with advanced disease or those in end-of-life care may have limited ability to self-assess HRQOL [12]

The aim of this study is to investigate patient-proxy HRQOL agreement in a sample of patients with recurrent HGG. Previous findings of a study conducted on low-grade glioma patients, in which part of the authors involved in the present study collaborated, suggest neurocognitive impairment as an influencing factor for HRQOL patient-proxy agreement. Therefore, we divided patients participating in two EORTC-coordinated clinical trials into neurocognitively impaired and intact, and used an approach similar to the one previously implemented in the study by Ediebah and colleagues. Our pre-trial hypothesis was that we expected neurocognitively impaired patients to have lower levels of patient-proxy agreement than neurocognitively intact patients [4–6, 13].

## Patients and methods

The initial sample of EORTC trials 26101 and 26091 combined consisted of 731 patients. Most patients had prior chemotherapy and radiotherapy, and in both trials, evaluation prior to randomization and every 12 weeks thereafter included neurocognitive, HRQOL, and full clinical assessment. However, in the present study, we exclusively used baseline data to determine patient-proxy agreement, not the change in agreement over time. This study is primarily interested in the level of agreement at a single point in time between proxy- and patient-reported HRQoL scores and aims to provide insight into how proxies evaluate patients' quality of life compared to the patients' own assessment. Future research will extend this analysis by evaluating how agreement evolves over time.

In addition to the criteria set for inclusion in the two clinical trials shortly described underneath, [14, 15] only HGG patients (i.e., WHO grade III and grade IV) were selected for this study. Since data had been collected prior to 2016, the 2007 WHO tumor classification was used, selecting only WHO grade III and IV tumors [16]. All variables were measured within two weeks prior to randomization. We determined a window of ± 7 days between neurocognitive functioning (NCF) evaluation and QLQ-C30 and QLQ-BN20 administration to ensure concurrent measurements, given the one-week time frame of the QLQ questions.

EORTC trial 26,101 (EudraCT number 2009–017422-39) was a randomized phase III trial investigating whether the combination of lomustine plus bevacizumab compared to lomustine alone would result in better overall survival in glioblastoma patients with first progression [14]. EORTC 26091 (EudraCT number 2010–023218-30) was a randomized, open-label phase II trial comparing temozolomide alone to the combination of temozolomide and bevacizumab in patients with a first recurrence of a locally diagnosed WHO grade II or III glioma without 1p/19q co-deletion [15].

### Ethics

These trials were approved by the institutional review boards and ethics committees of all participating centers and the respective authorities. The trials were completed according to the Declaration of Helsinki, and all patients provided written informed consent.

### Neurocognitive assessment

*Neurocognitive functioning (NCF)* was assessed using a widely accepted clinical trial battery for testing NCF in patients with intracranial or extracranial tumors selected because of their wide use in clinical trials and their sensitivity to the impact of tumor and treatment-related variables [17, 18]. This battery consists of the Hopkins Verbal Learning Test–Revised (HVLT-R) [19] for total recall, delayed recall, and delayed recognition, which indexes verbal learning and memory; the Trail Making Test (TMT part A and part B), [20] which measures attention, speed, and mental flexibility; and the Controlled Oral Word Association Test (COWAT), [21] which evaluates the spontaneous production of words under restricted search conditions. These tests were administered by centrally trained and certified health-care personnel, e.g., research nurses and neuropsychologists.

### Health-related quality of life assessment for patients

HRQOL was measured using the EORTC QLQ-C30 questionnaire [22] and the EORTC QLQ-BN20 module specific for brain tumor patients. [23] The former is a 30-items questionnaire developed to assess the quality of life of cancer patients. The latter is a 20-item module that tackles problems specific to brain tumors, their treatment, and consequences.

The QLQ-C30 is divided into functioning and symptom scales, while the QLQ BN20 is a symptom-only questionnaire. In functioning scales, the higher the scores, the better the functioning, and in symptom scales, the opposite is true; a higher score indicates more of the symptoms.

HRQOL questionnaires were filled out at the hospital when patients had scheduled visits. Patients completed the questionnaire in the clinic, ideally in a quiet, private room; questionnaires were given to the patient before meeting with the physician, ensuring that the patient had enough time to complete the questionnaire. If the patient received therapy, the questionnaire was filled out before the administration of the treatment. The questionnaire could not be taken home and/or mailed.

### Health-related quality of life assessment for proxies

Consenting patients were requested to identify a significant other (i.e., the spouse or other person in a close relationship to the patient), whom physicians asked to participate in the study. The significant others were also provided with verbal and written information on the study.

Patients’ proxies were asked to complete the EORTC QLQ-C30 and EORTC QLQ-BN20 at each assessment point at the same time as the patient, at baseline and at 12-weekly follow-ups. Proxies were also instructed to complete the questionnaire, trying to put themselves in the shoes of the patients since the questions were formulated always in first person. During the design phase of trials 26101 and 26091, it was considered counterproductive to collect demographic data on the proxies. This would have necessitated obtaining informed consent from the proxies, potentially reducing recruitment rates for these EORTC studies.

## Statistical analysis

Descriptive statistics of the sample were calculated, with mean and standard for continuous data and count and/or percentages for binary data. For each of the six NCF outcome measures (1) HVLT-R Total Recall, (2) HVLT-R Delayed Recall, (3) HVLT-R Delayed Recognition, (4) TMT Part A, (5) TMT Part B, and (6) COWA, raw scores (RS) were calculated [19–21].

Raw scores of the six NCF test outcomes were transformed into Z-scores using available normative data. [19–21] A deviation of -1.5 SD or more from the Z-score mean was used as a cutoff to define NCF impairment. Furthermore, a sensitivity analysis raising the SD from 1.5 to 2 was performed to show if agreement was influenced by the arbitrary definition of impairment adopted in the present study and can be found as an appendix to the present study (Supplemental Material). Based on the presence of impaired test outcomes, patients were consecutively divided into two groups. Patients who had no impairment on any of the six test outcomes were defined as ‘intact’, while patients showing at least one impaired test were defined as ‘impaired’. [24]“Due to the definition of intact patients being those without any NCF deficits, only patients with all six NCF outcome measures available were included in the statistical analyses. (See supplemental material for comparison analysis with excluded patients.)”.

The QLQ-C30 and QLQ-BN20 are questionnaires based on a Likert scale answer system, and multi-item and single-item subscales are formed, addressing general functioning as well as symptoms. A higher score on a functioning scale corresponds to better functioning, and a higher score on a symptom scale corresponds to more symptoms.

Patients with more than half of the indices of neurocognitive functioning (NCF), EORTC Quality of Life Core Questionnaire (QLQ-C30), or Quality of Life Brain Module (QLQ-BN20) evaluations unavailable were excluded from the analyses.

QLQ-C30 and QLQ-BN20 raw scores were transformed into a linear scale ranging from 0 to 100 [25]. Mean differences and standard deviations between patients and proxy were calculated. The proportion of patient-proxy dyads whose difference was within 0, 10,20, and more than 20 units was summarized. Then, scores of patients and proxies on all QLQ-C30 and QLQ-BN20 scales were compared using Lin’s concordance correlation coefficient (CCC) and the Bland–Altman limit of agreement. The Wilcoxon signed-rank test was used to evaluate differences between patients’ and proxies’ HRQOL.

The Wilcoxon signed-rank test was used to compare the distributions of the patients and proxies scores, looking for differences and, more importantly, to identify eventual systematic bias. Such bias can be caused, for instance, by a higher median for proxy scores compared to patient scores.

The Bland–Altman indicates the range within which 95% of all differences in ratings are expected to fall, assuming distribution normality. It was implemented to compare patient-proxy agreement by offering plausible ranges for differences in scores. [26–28]

Lastly, Lin’s Concordance Correlation Coefficient (CCC) was used to evaluate the concordance between patient and proxy ratings. A score below 0.40 indicated poor to fair agreement; 0.41–0.60, moderate agreement; 0.61–0.80, good agreement; 0.81–1.00, excellent agreement [26].

The reason why we decided to implement multiple measures to assess patient-proxy agreement is to compensate for the limits of each individual technique. Regarding Lin’s CCC, if proxies were to consistently overestimate or underestimate HRQOL compared to patients, the CCC may not effectively capture this directional bias by emphasizing overall correlation rather than specific deviations. The Bland–Altman method operates on the assumption that the differences between measurements are normally distributed, which in patient-reported outcomes such as the HRQOL may not always be the case. To avoid the shortcomings of both techniques, we decided to resort to the Wilcoxon signed rank test, which allows for comparison between paired observations without assuming normal distribution.
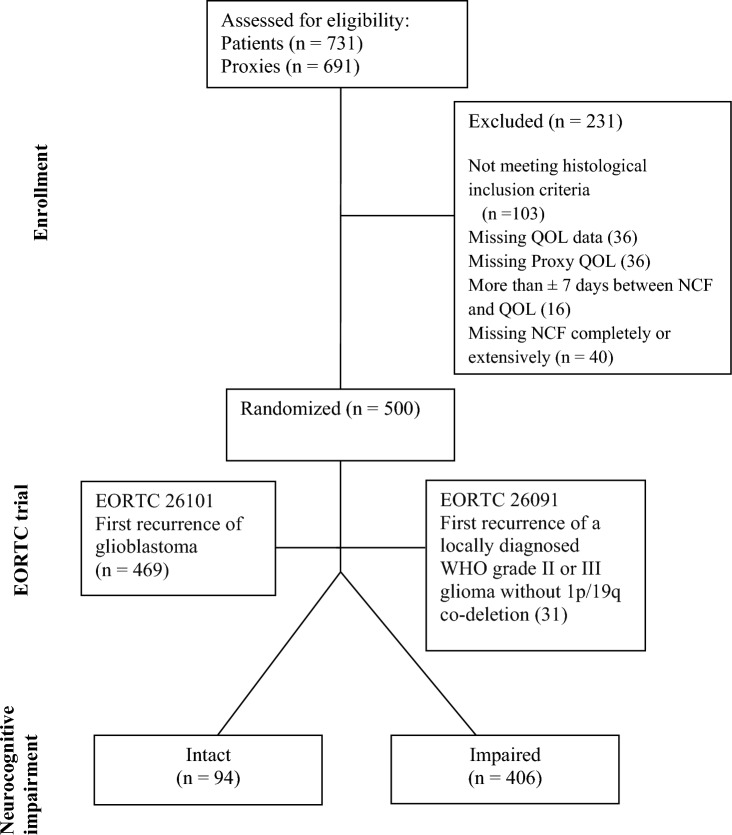


## Results

From the initial cohort of 731 patients and 691 proxies, 103 patients did not meet the histological criteria of WHO grade III and IV high-grade gliomas, 40 patients had incomplete NCF evaluations, 36 patients were missing HRQOL evaluations, 36 patients were missing HRQOL filled by proxies, and 16 patients had HRQOL and NCF evaluations measured more than ± 7 days apart from each other. After the exclusion of those patients with extensively missing data and their significant others together with them, a total of 500 patients were selected: 469 with first recurrence of glioblastoma (EORTC 26101) and 31 patients with first recurrence of a locally diagnosed WHO grade II or III glioma without 1p/19q co-deletion (EORTC 26091). Patients included for the current analyses (*n* = 500) had a median age of 56 years with a range of 21–82, and 183 (36.6%) were female. Further detailed clinical information can be found in Table 1.

**Table 1:** Baseline Clinical Characteristics

Baseline clinical characteristics					
		N°	%		
Age	Median = 56, Range = (21—82)				
Gender					
	Female	183	36.6		
	Male	317			
WHO Performance Status					
	0	175	35		
	1	271	54.2		
	2	54	10.8		
Histology					
	Glioblastoma	435	87		
	Astrocytoma WHO grade III	30	6		
	Glioblastoma with Oligodendroglial component	21	4.2		
	Gliosarcoma	8	1.6		
	Giant Cell Glioblastoma	3	0.6		
	Missing/Unknown	3	0.6		
Tumor location					
		Hemisphere			
		Left	Bilateral	Right	
	Total	210	23	232	
	Frontal	60	5	65	26
	Temporal	72	0	79	30.2
	Parietal	16	0	20	7.1
	Occipital	20	1	22	8.6
	Other	19	16	10	9
	Multi-site	23	1	366	12
	Missing	35			7
AED (Anti-epileptic drugs)					
	Yes	332			67.2
	No	167			33.4
	Missing	1			0.2
Corticosteroids					
	Yes	250			50
	No	250			50
Neurocognitive Impairment					
	Intact	94			18.8
	Impaired	406			81.2

WHO (World Health Organization), AED (Anti-epileptic drugs)

### Agreement between patients and proxies

Table 2 summarizes the QLQ-C30 and QLQ-BN20 outcomes for all 500 patients meeting the inclusion criteria, regardless of their neurocognitive status.

**Table 2:** Patient-proxy agreement measured by Lin’s CCC and Wilcoxon sign ranked test for the whole sample

All Patients	Mean Proxy	Mean Patients	Mean Difference (Proxy–Patient) (SD)	% Points within the 95% limit of agreement (LL-UL)	% Within 0 points		% With < 10	% With < 20	% With > 20	Missing	CCC (95% CI)	*Wilcoxon *p* values*
QLQ-C30												
Global Health	62.45	62.77	− 0.25 (21.17)	94 (41.24–41.75)	25.2		25.4	22.8	25.6	1.0	.499 (.430–.563)	0.949
Physical Functioning	73.90	76.85	− 2.90 (17.17)	94.4 (30.74–36.55)	31.2		30.2	21.4	16.8	0.4	.743 (.698–.781)	0.001
Role Functioning	62.00	63.99	− 1.82 (27.18)	95 (51.46–55.1)	41.4		0,00	28.4	29.4	0.8	.641 (.586–.690)	0.075
Emotional Functioning	61.83	68.11	− 6.18 (20.67)	95.8 (34.34–46.7)	22.4		27.4	18.6	30.8	0.8	.604 (.520–.673)	0.001
Cognitive Functioning	63.16	67.10	− 3.81 (24.22)	95 (43.66–51.28)	36.6		0,00	36.4	26.2	0.8	.612 (.551–.665)	0.001
Social Functioning	65.45	67.81	− 2.04 (27.86)	95.2 (52.57–56.65)	37.8		0,00	30.4	30.8	1.0	.579 (.518–.635)	0.171
Fatigue	41.61	36.36	5.14 (22.08)	94.6 (38.15–48.42)	30.0		0.8	31.4	37.2	0.6	.612 (.544–.671)	0.001
Nausea and Vomiting	3.42	4.58	− 1.18 (11.42)	94.4 (21.21–23.57)	81.0		0,00	12.8	5.6	0.6	.408 (.332–.479)	0.009
Pain	16.73	14.83	1.9 (21.24)	92.2 (39.73–43.53)	56.0		0,00	24.8	19.2	0,00	.600 (.541–.654)	0.033
Dyspnoea	11.76	12.22	− 0.61 (21.63)	96 (41.79–43.01)	60.2		0,00	37.6	0,00	2.2	.491 (.421–.555)	0.543
Insomnia	28.70	26.41	2.03 (26.92)	93.4 (50.73–54.79)	59.2		0,00	0,00	39.6	1.2	.612 (.554–.665)	0.114
Appetite loss	8.52	9.86	− 1.35 (19.98)	97 (37.82–40.52)	76.6		0,00	0.2	22.6	0.6	.490 (.420–.554)	0.141
Constipation	12.37	12.50	− 0.14 (18.77)	97.6 (36.65–36.93)	74.6		0,00	0.6	21.8	3.0	.678 (.627–.724)	0.888
Diarrhoea	6.15	4.64	1.54 (17.12)	92.06 (32–35.09)	82.8		0,00	0.2	13.6	3.4	.399 (.321–.472)	0.053
Financial difficulties	20.12	17.61	2.12 (31.39)	91.8 (59.41–63.65)	64.6		0,00	0.6	33.4	1.4	.470 (.398–.536)	0.332
QLQ BN-20												
Future uncertainty	44.38	42.44	1.95 (23.87)	93.2 (44.83–48.73)		16.2	28.0	20.8	34.2	0.8	.586 (.525–.641)	0.137
Visual disorders	15.07	16.35	− 1.22 (16)	92.2 (30.13–32.58)		46.6	1	30.6	20.4	1.4	.675 (.624–.721)	0.127
Motor dysfunction	23.54	20.88	2.81 (19.02)	94.2 (34.47–40.09)		39.0	0.4	30.2	29.8	0.6	.705 (.657–.748)	0.002
Communication deficit	26.77	24.61	2.20 (20.62)	92.4 (38.22–42.62)		39.8	0.2	28.2	30.8	1.0	.725 (.680–.765)	0.015
Headache	20.08	19.99	− 0.21 (22.12)	96.6 (43.15–43.57)		68.8	0,00	1.2	28.0	2.0	.671 (.619–.717)	0.663
Seizures	4.75	5.73	− 0.90 (16.15)	88.6 (30.75–32.55)		86.8	0,00	0.4	11.4	1.4	.508 (.439–.571)	0.288
Drowsiness	29.87	26.50	3.72 (28.21)	92 (51.58–59.01)		52.4	0,00	2.0	44.4	1.2	.505 (.435–.568)	0.003
Hair loss	10.86	9.63	0.91 (21.45)	95 (41.13–42.95)		74.8	0,00	1.0	21.4	2.8	.563 (.498–.621)	0.289
Itchy skin	7.78	10.62	− 2.64 (21.59)	95.2 (39.68–44.96)		76.0	0,00	0.8	21.4	1.8	.453 (.379–.522)	0.009
Weakness in legs	16.91	14.53	2.43 (24.64)	94.2 (45.52–50.37)		66.8	0,00	0.8	30.8	1.6	.523 (.455–.585)	0.036
Bladder control	9.44	11.67	− 2.12 (17.94)	90.8 (33.04–37.28)		79.4	0,00	0.6	19.2	0.8	.686 (.636–.731)	0.011

% 0 (cases of no difference between patient- proxy evaluation); % < 10 (cases with less than 10 points of difference between patient-proxy evaluation); % < 20 (cases with less than 20 points of difference between patient- proxy evaluation); % > 20 (cases with more than 20 points of difference between patient-proxy evaluation);

CCC (Concordance Correlation Coefficient); LL (Lower limit); UL (Upper limit); C.I (Confidence interval)

Differences in scores of patients and proxies were observed on various functioning and symptom scales, with patients reporting a higher score (better functioning/more symptoms) than their proxies on Physical Functioning, Emotional functioning, Cognitive functioning and Nausea and Vomiting, scales of the QLQ-C30 and on Itchy skin and Bladder control scales of the QLQ-BN20.

The opposite held true, with patients reporting lower scores (worse functioning/less symptoms) than their proxies on Fatigue and Pain scales of the QLQ-C30 and Motor dysfunction, Communication deficit, Drowsiness and Weakness in legs scales of the QLQ BN20.

Lin’s CCC showed moderate to good agreement ranging from r = 0.399, 95% CI (0.321—0.472) (Diarrhea) to r = 0.743, 95% CI (0.698—0.781) (Physical functioning).

Lastly, the Bland–Altman limit of agreement showed agreement between patients and proxies in the Role Functioning, Emotional Functioning, Cognitive Functioning, Social Functioning, Dyspnea, Appetite loss, Constipation, Headache, Hair loss and Itchy skin domains.

The difference between patients and proxies was calculated, and the proportion within 0 (perfect agreement) and more than 20 units was summarized with a range of 16.2% (Future uncertainty) to 86,8% (Seizures) and 0% (Dyspnea) to 44.4% (Drowsiness) respectively.

### HRQOL agreement between neurocognitively impaired patients and proxies

In total, 94 patients were neurocognitively intact, while 406 were impaired according to our definition. Differences in scores of neurocognitively impaired patients and their proxies were observed on some of the functioning and symptoms scales, with patients reporting a higher score than their proxies on the Physical functioning, Emotional functioning, Cognitive functioning, and Nausea and vomiting scales of the QLQ-C30.

On the other hand, neurocognitively impaired patients reported lower scores than their proxies on the Fatigue and Diarrhea scales of the QLQ-C30 and on the Motor Dysfunction, Communication deficit, Drowsiness, and Weakness in legs scales of the QLQ BN20.

Lin’s CCC showed fair to moderate agreement, ranging from r = 0.376, 95% CI (0.288—0.458) (Diarrhea) to r = 0.732, 95% CI (0.682—0.775) (Physical functioning).

The Bland–Altman limit of agreement revealed agreement between patients and proxies in the Physical Functioning, Role Functioning, Emotional Functioning, Cognitive Functioning, Social Functioning, Fatigue, Nausea and Vomiting, Dyspnea, Appetite loss, Constipation, and Diarrhea domains on the QLQ-C30 and Communication deficit, Headache, Seizures, Hair loss, Itchy skin, and Bladder control on the QLQ BN20.

The difference between patients and proxies was calculated, and the proportion within 0 and more than 20 units was summarized with a range of 16.7% (Future uncertainty) to 86.9% (Seizures) and 0% (Dyspnea) to 46.3% (Drowsiness) respectively.

Table 3 shows the scores of neurocognitively impaired patients and their proxies.

**Table 3:** Patient-proxy agreement measured by Lin’s CCC and Wilcoxon sign ranked test for neurocognitively Impaired patients

Impaired Patients	Mean Proxy	Mean Patients		Mean Difference (Proxy—Patient) (SD		% Points within the 95% limit of agreement (LL-UL)	% Within 0 points		% With < 10		% With < 20		% With > 20		Missing		CCC (95% CI)	*Wilcoxon *p* values*
QLQ-C30																		
Global Health	60.48	60.70	− 0.15 (21.17)		92.6 (41.35–41.64)		25.1	23.7		23.1		27.1		1.0		.498 (.421–.569)		0.889
Physical Functioning	71.40	74.35	− 2.92 (18.02)		95 (32.39–38.23)		29.6	30.3		21.7		18.3		0.2		.732 (.682–.775)		0.001
Role Functioning	58.81	61.28	− 2.32 (28.05)		95.4 (52.67–57.32)		39.2	0,00		28.6		31.5		0.7		.626 (.523–.682)		0.061
Emotional Functioning	60.03	67.06	− 7.00 (21.17)		96.6 (34.5–48.5)		22.2	26.1		17.5		33.7		0.5		.601 (.502–.680)		0.001
Cognitive Functioning	59.58	63.90	− 4.24 (25.28)		95.4 (45.3–53.78)		34.5	0,00		36.7		28.1		0.7		.589 (.519–.651)		0.002
Social Functioning	62.26	64.97	− 2.47 (28.93)		95.4 (54.23–59.18)		36.9	0,00		29.3		33.0		0.7		.564 (.493–.627)		0.134
Fatigue	43.92	37.90	5.96 (22.52)		95.4 (38.18–50.09)		29.1	0.7		31.0		38.9		0.2		.603 (.522–.670)		0.001
Nausea and Vomiting	3.54	4.86	− 1.32 (11.37)		95.2 (20.96–23.6)		81.0	0,00		12.8		5.9		0.2		.438 (.356–.514)		0.010
Pain	17.65	15.89	1.77 (22.11)		93.2 (41.56–45.09)		53.9	0,00		25.9		20.2		0,00		.585 (.518–.646)		0.087
Dyspnoea	13.02	13.33	− 0.42 (22.79)		96.6 (44.25–45.08)		58.9	0,00		39.4		0,00		1.7		.477 (.398–.549)		0.737
Insomnia	29.05	26.12	2.74 (27.60)		94.2 (51.36–56.84)		58.6	0,00		0,00		40.7		0.7		.595 (.527—.654)		0.056
Appetite loss	9.08	10.81	− 1.74 (20.52)		97.2 (38.47–41.95)		75.9	0,00		0,00		23.7		0.5		.503 (.426–.572)		0.109
Constipation	12.35	12.13	0.26 (18.55)		98 (36.11–36.63)		82.3	0,00		0.2		14.8		2.7		.669 (.610–.721)		0.752
Diarrhoea	6.77	4.80	1.98 (18.09)		98 (33.47–37.43)		62.6	0,00		0.7		35.5		1.2		.376 (.288–.458)		0.032
Financial difficulties	20.25	19.25	0.59 (29.42)		93.2 (58.25–57.07)		62.6	0,00		0.7		35.5		1.2		.515 (.439–.584)		0.866
QLQ BN-20																		
Future uncertainty	45.71	43.85	1.93 (24.34)		94.4 (45.78–49.65)		16.7	26.1		20.7		36.0		0.5		.584 (.515–.645)		0.189
Visual disorders	16.68	17.65	− 0.87 (16.72)		94.2 (31.89–33.64)		43.3	1.0		31.5		22.9		1.2		.668 (.610–.719)		0.350
Motor dysfunction	26.42	22.96	3.63 (19.97)		94.6 (35.51–42.77)		36.2	0.5		30.0		32.8		0.5		.690 (.632–.740)		0.001
Communication deficit	30.88	27.63	3.25 (21.69)		95.8 (39.26–45.75)		36.0	0.2		29.5		33.3		1.0		.712 (.659–.758)		0.002
Headache	20.65	20.89	− 0.26 (22.61)		97 (44.06–44.57)		68.7	0,00		1.2		28.1		2.0		.670 (.611–.721)		0.638
Seizures	5.12	6.03	− 0.77 (16.80)		95.6 (32.16–33.69)		86.9	0,00		0.2		11.3		1.5		.523 (.447–.591)		0.459
Drowsiness	32.16	28.35	4.24 (29.43)		92.4 (53.45–61.93)		50.5	0,00		2.2		46.3		1.0		.479 (.399–.522)		0.004
Hair loss	11.37	10.13	1.04 (22.54)		95.4 (43.14–45.22)		73.2	0,00		1.2		22.6		3.0		.541 (.466–.608)		0.312
Itchy skin	8.40	10.23	− 1.71 (21.42)		96 (40.27–43.68)		76.4	0,00		0.7		21.5		1.5		.453 (.371–.528)		0.122
Weakness in legs	18.94	15.79	3.25 (25.87)		94.6 (47.46–53.95)		64.0	0,00		0.7		33.8		1.5		.497 (.418–.568)		0.016
Bladder control	10.16	12.79	− 2.53 (19.13)		97.2 (34.96–40.02)		77.6	0,00		0.7		20.9		0.7		.666 (.607–.718)		0.010

% 0 (cases of no difference between patient- proxy evaluation); % < 10 (cases with less than 10 points of difference between patient-proxy evaluation); % < 20 (cases with less than 20 points of difference between patient- proxy evaluation); % > 20 (cases with more than 20 points of difference between patient-proxy evaluation)

CCC (Concordance Correlation Coefficient); LL (Lower limit); UL (Upper limit); C.I (Confidence interval)

### HRQOL agreement between neurocognitively intact patients and proxies

The analysis of the scores of neurocognitively intact patients and their proxies showed significant differences on two functioning and symptoms scales, with patients reporting a higher score than their proxies on the Visual disorder and the Itchy skin scale of the QLQ-BN20.

Lin’s CCC ranged from poor to good with the lowest agreement on the Nausea and vomiting scale, r = 0.231, 95% CI (0.027–0.416), and the highest on the Bladder control scale, r = 0.811, 95% CI (0.728–0.871).

The Bland–Altman limit of agreement revealed agreement between patients and proxies on all functioning and symptom scales of the QLQ-C30 and QLQ BN20.

The difference between patients and proxies was calculated, and the proportion within 0 and more than 20 units was summarized with a range of 13.8% (Future uncertainty) to 87. % (Bladder control) and 4.2% (Nausea and vomiting) to 36.2% (Drowsiness) respectively. See Table 4.

**Table 4:** Patient-proxy agreement measured by Lin’s CCC and Wilcoxon sign ranked test for neurocognitively Intact patients

Intact Patients	Mean Proxy	Mean Patients		Mean Difference (Proxy—Patient) (SD		% Points within the 95% limit of agreement (LL-UL)	% Within 0 points		% With < 10		% With < 20		% With > 20		Missing	CCC (95% CI)	*Wilcoxon *p* values*
QLQ-C30																	
Global Health	70.97	71.68	− 0.72 (21.28)		99 (40.98–42.23)		25.5	33.0		22.3		18.0		1.1		.377 (.187–.540)	0.860
Physical Functioning	84.82	87.64	− 2.83 (12.9)		98.6 (22.46–28.12)		38.3	29.8		20.2		10.7		1.1		.717 (.600–.803)	0.1
Role Functioning	75.81	75.71	0.36 (23.05)		98.4 (44.82–45.54)		51.1	0,00		27.5		20.2		1.1		.641 (.503–.747)	0.972
Emotional Functioning	69.66	72.61	− 2.63 (18.03)		98.8 (32.71–37.96)		23.4	33.0		23.4		18.0		2.1		.585 (.433–.704)	0.090
Cognitive Functioning	78.67	80.85	− 1.97 (19.01)		99.2 (35.29–39.23)		45.7	0,00		35.1		18.0		1.1		.559 (.402–.684)	0.304
Social Functioning	79.35	79.96	− 0.18 (22.71)		98.4 (44.33–44.7)		41.5	0,00		35.1		21.2		2.1		.545 (.383–.674)	0.953
Fatigue	31.40	29.69	1.53 (19.77)		99.4 (37.23–40.28)		34.0	1.1		33.0		29.8		2.1		.611 (.464–.725)	0.405
Nausea and Vomiting	2.90	3.37	− 0.54 (11.71)		99.2 (22.4–23.49)		80.9	0,00		12.8		4.2		2.1		.231 (.027–.416)	0.497
Pain	12.77	10.28	2.48 (17.1)		98.4 (31.04–36)		64.9	0,00		20.2		14.9		0,00		.668 (.540–.766)	0.150
Dyspnoea	6.23	7.45	− 1.47 (15.64)		97.8 (29.2–32.13)		66.0	0,00		29.8		4.3		0,00		.550 (.389–.678)	0.377
Insomnia	27.11	27.66	− 1.1 (23.54)		99.2 (45.05–47.25)		61.7	0,00		0,00		35.1		3.2		.698 (.575–790)	0.639
Appetite loss	6.09	5.73	0.36 (17.47)		99.8 (33.87–34.6)		79.8	0,00		1.1		18.1		1.1		.363 (.171–.528)	1.000
Constipation	12.45	14.13	− 1.87 (19.69)		99.6 (36.73–40.47)		70.2	0,00		0,00		26.6		3.2		.712 (.593–.801)	0.364
Diarrhoea	3.41	3.94	− 0.38 (11.85)		98.4 (22.84–23.6)		85.1	0,00		0,00		8.6		6.4		.562 (.400–.690)	0.763
Financial difficulties	19.57	10.64	8.7 (28.24)		99 (66.25–83.64)		73.4	0,00		0,00		24.5		2.1		.313 (.121–.483)	0.051
QLQ BN-20																	
Future uncertainty	38.65	36.35	2.02 (21.81)		98.4 (40.72–44.76)		13.8	36.2		21.3		26.6		2.1		.572 (.417–.695)	0.483
Visual disorders	8.18	10.75	− 2.72 (12.42)		99 (21.63–27.07)		60.6	1.1		26.5		9.6		2.1		.671 (.541–770)	0.033
Motor dysfunction	11.23	11.83	− 0.72 (13.79)		98.6 (26.3–27.74)		51.1	0,00		30.9		17.0		1.1		.715 (.599–.801)	0.476
Communication deficit	9.22	11.59	− 2.27 (14.52)		98.8 (26.19–30.73)		56.4	0,00		22.4		20.2		1.1		.618 (.475–.729)	0.163
Headache	17.58	16.13	0 (19.99)		99.6 (39.18–39.18)		69.1	0,00		1.1		27.6		2.1		.673 (.541–.772)	1.000
Seizures	3.19	4.40	− 1.47 (13.06)		97.8 (24.14–27.07)		86.2	0,00		1.1		11.7		1.1		.363 (.171 –528)	0.285
Drowsiness	20.07	18.48	1.47 (22.17)		99.6 (41.99–44.92)		60.6	0,00		1.1		36.2		2.1		.588 (.435–.707)	0.528
Hair loss	8.70	7.53	0.37 (16.1)		97 (31.18–31.92)		81.9	0,00		-		15.9		2.1		.688 (.562–783)	0.806
Itchy skin	5.13	12.32	− 6.74 (22.01)		99.2 (36.39–49.88)		74.5	0,00		1.1		21.3		3.2		.464 (.281–.614)	0.005
Weakness in legs	8.24	9.06	− 1.1 (16.81)		98.4 (31.86–43.06)		78.7	0,00		1.1		18.0		2.1		.648 (.511–753)	0.536
Bladder control	6.38	6.81	− 0.36 (11.52)		97.8 (22.22–22.94)		87.2	0,00		0,00		11.7		1.1		.811 (.728–871)	0.763

% 0 (cases of no difference between patient- proxy evaluation); % < 10 (cases with less than 10 points of difference between patient-proxy evaluation); % < 20 (cases with less than 20 points of difference between patient- proxy evaluation); % > 20 (cases with more than 20 points of difference between patient-proxy evaluation)

CCC (Concordance Correlation Coefficient); LL (Lower limit); UL (Upper limit); C.I (Confidence interval)

## Discussion

Measuring neurocognitive functioning is essential in brain tumor patients because this may not only influence HRQOL but also patient–proxy concordance levels. Although the patient’s responses are inherently considered a true source of information when measuring HRQOL, the information collected from patients with glioma may be unreliable, especially in those patients who are experiencing significant neurocognitive deterioration [7]. Therefore, obtaining proxy HRQOL ratings alongside patient-reported outcomes is paramount because it allows for substituting patient-by-proxy ratings when a patient’s self-report is absent.

In the present study, we aimed at assessing patient-proxy HRQOL agreement in a sample of recurrent HGG patients with and without neurocognitive impairment. To achieve this, we compared the baseline scores of patients and proxies from the EORTC trial 26101 and 26091 on the QLQ-C30 and QLQ-BN20 questionnaires.

We found that there was overall moderate agreement between the patient and patient-by-proxy rating of HGG patients HRQOL in most subscales of the QLQ-C30 and QLQ-BN20 when not considering neurocognitive impairments. The statistically significant differences were Physical Functioning, Emotional functioning, Cognitive functioning, Fatigue and Pain and Nausea and Vomiting, scales of the QLQ-C30 and on Motor dysfunction, Communication deficit, Drowsiness, Itchy skin, Weakness in legs and Bladder control scales of the QLQ-BN20.

Agreement between patients and patient-by-proxy rating of HGG neurocognitively impaired patients HRQOL in most subscales of the QLQ-C30 and QLQ-BN20 showed significant differences in the same scales as for the whole group, except for the Itchy skin subscale replaced by the diarrhea scale. The only significant differences in the neurocognitively healthy group were found in the visual disorder and itchy skin scales of the QLQ-BN20.

The difference between patient and patient-by-proxy ratings found in the whole group of patients could be due to the presence of cognitively impaired patients. The fact that the differences in agreement on many scales were not statistically significant when investigating neurocognitive intact patients alone seems to support this idea. However, it is not possible to exclude the possibility that significant differences were influenced by the difference in sample size between cognitively intact and impaired patients. Furthermore, we were expecting to observe more differences in functioning and symptom scales describing non-observable symptoms, and this is somehow supported by the noticeably largest differences observed in mean scores of scales such as Emotional functioning, Cognitive functioning, fatigue, and drowsiness, both in all patients and neurocognitively impaired ones. However, these differences were not as evident when looking at the Bland–Altman limit of agreement and Lin’s CCC. One hypothesis that could explain this phenomenon is related to what has been shown by Giesinger and colleagues, who reported how potential discrepancies in agreement between patient and proxy ratings are minimized by the limited range scale of the tool used [29]. In this case the scoring system of the QLQ C30 and QLQ BN20 might influence the agreement due to the necessity of transforming the scores from a Likert scale to a 0–100 score.

These results are in line with other studies on patient-proxy agreement in brain cancer patients. [7, 30] When looking at the general agreement of HGG patients with their proxies and comparing it to the results published by Brown and colleagues in a similar population and, by Sneeuw and colleagues, on a generic cancer population, the agreement reported in our sample is comparable. Using a similar statistical approach, the first study reported an ICC between patients and proxies greater than 0.5 on 80% of the measurements; the second showed ICCs ranging from 0.46 to 0.73, indicating a moderate to good level of agreement between patients and proxies. In our study, the agreement ranged from 0.39 to 0.74, with almost all scales between 0.4 and 0.8 thresholds that indicate moderate agreement.

According to the literature [4, 6, 23, 24], neurocognitive impairment affects patient-proxy agreement. Therefore, we expected neurocognitive impairment to be an influencing factor. Altogether, the findings of the present study replicate the literature results suggesting a certain degree of difference between cognitively impaired and intact patients. However, using different statistical methods to assess the level of agreement forces us to consider the matter from different angles. While the differences in mean scores and the Wilcoxon signed rank test coefficients stand for a clear difference between the two groups of patients, the results of Lin’s CCC and Bland–Altman analysis do not offer an equally clear boundary.

There are some aspects that could have played a role in this lack of consistency. The sample of patients investigated in this sample is characterized by tumor recurrence, and while proxies may not fully realize the emotional impact of declining intellectual function in patients with newly diagnosed tumors, the proxies analyzed in this study might carry some levels of deeper insight. Furthermore, there are other aspects that were not accessible for the present studies but that have been reported in Alzheimer disease (AD) literature to play a role in patient-proxy agreement that mostly relate to the proxy figure, such as proxies’ cognitive skills, valuation of life, and level of depression and anxiety that may influence the accuracy of the caregiver report. [31–33]

It is important to mention that there are several limitations to this study:

The Bland–Altman limit of agreement does not capture certain aspects of the data, particularly in the context of health-related quality of life (HRQOL) assessments where subjective elements and varying perspectives are involved. While recognizing that there are several statistical methods to evaluate agreement between raters and that they are designed for different data types and have key conceptual differences, underlying assumptions, and calculation methods, we decided to employ the Wilcoxon signed-rank test to measure HRQOL rating discrepancies between patients and their proxies and Lin’s Concordance Correlation Coefficient to measure agreement. Data concerning the level of kinship of proxies was not recorded at the EORTC headquarters. At the time of the design of these studies, it was regarded as counterproductive to register demographic data on the proxies, as that would have required informed consent by the proxy as well, possibly negatively influencing recruitment rates of these EORTC studies. No information about specific procedures used to ensure tests were independently completed was recorded. However, in each institution, one person was appointed as responsible for the local organization of HRQOL data collection. This could have been a physician, data manager, (research) nurse, or psychologist.

Furthermore, no direct measure of mood, which has been shown to offer even more insight into patient-proxy levels of agreement, was collected [8, 34]. The percentage of mean differences between dyads (0, 10, 20, or more than 20) might have been influenced by the number of possible scores on a scale. [23] The fact that treatment course and disease duration prior to inclusion may have been different between patients in the two trials might have impacted sample homogeneity. While it is true that most of the sample (93.8%) was diagnosed with Glioblastoma, there was heterogeneity among the patients. *Our definition of neurocognitive impairment based on the number of available test outcomes is somewhat arbitrary, and the decision to perform complete case analysis, excluding those patients with missing cognitive evaluations, limits generalizability of our findings. A comparative analysis between the neurocognitively impaired patients included in the study and 37 patients that were excluded due to missing NCF outcomes demonstrated how included patients had favorable outcome in three of the six NCF outcomes. This indicates that generalizability is limited to relatively well-functioning patients. Detailed information can be found in the supplemental material.* Furthermore, it is possible that by grading the extent of neurocognitive impairment in levels rather than in a dichotomic variable, results might be different. Unfortunately, this was not possible. Nevertheless, a sensitivity analysis raising the threshold for neurocognitive dysfunction per test (> 2 SD) was performed (Supplemental Material). By exacerbating the definition of neurocognitive impairment from what is commonly considered the impairment threshold in the clinical environment, we hoped to include only those with an impaired performance even if on only one of the NCF tests. Results show how raising the neurocognitive impairment threshold can influence the statistical aspects related to differences in mean between patients and their proxies; it does not influence the general level of agreement between them, and therefore the threshold adopted in the present study does not limit its message.

The aim of this study was to assess patient-proxy HRQOL agreement in a sample of recurrent HGG patients with and without neurocognitive impairment. To our knowledge, this paper is the only one in the literature investigating patient-proxy agreement in glioblastoma in a large sample and using the QLQ C30 and the QLQ BN20. We are aware of other publications regarding patients affected by different kinds of brain tumor, however, considering the symptomatology of this specific kind of tumor in concurrence with the differences in quality-of-life tests adopted in different parts of the world, we believe that the present study can offer a significant contribution to the body of literature investigating the topic.

If it is true that patient-by-proxy ratings can help address compliance issues when assessing HRQOL in glioma patients with intact neurocognitive function, they can be even more useful when patients face cognitive impairment and lack the ability or insight to interpret and evaluate HRQOL measures.

In the current study, our expectations were partially met since we observed moderate agreement between patient and patient-by-proxy ratings for the entire sample and lower agreement for patients with impaired neurocognitive function compared to those with intact function. While patient and patient-by-proxy ratings in such cases should not be automatically considered incorrect, it is important to understand the sources of variation between these ratings.

The intrinsic subjectivity of health-related quality of life evaluation makes it difficult to establish what the ‘truth’ is. Our initial assumption was based on a syllogism for which a cognitively intact patient could be considered a reliable source of his/her own quality of life and a caregiver should be a reliable observer, at least for those scales describing functioning aspects and observable symptoms.

We are also aware that, because of their subjective nature, patient-reported outcomes (PROs) by definition are regarded as inherently accurate. However, the question that would follow is a predictable one: Would it be legitimate to not rely on a patient’s evaluation of his/her own well-being due to neurocognitive impairment?

Mental incapacity is frequently observed in patients with intracranial tumors and cognitive impairment is linked to this incapacity; yet clinicians often underestimate the patient’s limited capacity not only when it comes to patient-reported outcomes but even when obtaining consent for neuro-oncologic treatment [35].

The results of this study suggest that the moderate level of patient-proxy agreement observed in HGG patients would allow reliance on the proxy’s report. However, the differences observed between neurocognitively impaired and intact patients stress the importance of taking into consideration patients’ clinical and neurocognitive status as well as their mental capacity for adequate clinical decision-making in general and for PRO-related issues in particular.

## Supplementary Information

Below is the link to the electronic supplementary material.

Supplementary file1 (DOCX 47 KB)

## Data Availability

Study data are available upon reasonable request.
