# Supplementary material for: Neurocognitive impairment and patient-proxy agreement on health-related quality of life evaluations in recurrent high-grade glioma patients
Source: Qual Life Res. 2025 Jun 5;34(8):2405–18. doi: 10.1007/s11136-025-03984-1 (PMC12274216; doi:10.1007/s11136-025-03984-1)
Supplement: Supplementary file 1 — Supplementary file1 (DOCX 47 KB) [file 11136_2025_3984_MOESM1_ESM.docx]

**Sensitivity analysis**

Sensitivity analysis was performed raising the threshold to consider a patient as neurocognitively impaired: at least one out of six neurocognitive tests had to have 2.0 SD of difference from the standardized score. Mean scores for patient ad proxies and the mean difference between them was calculated. Patient-proxy agreement was calculated using Lin’s CCC and scores were tested with Wilcoxon signed rank test to determine significative difference between the assessments of the dyads.

In Table 1 and 2 is possible to compare the outcomes of the statistical tests mentioned above and compare it with those performed in our study.

With a raised threshold of 2 SD, Intact patients changed from 94 to 142 while Impaired patients decreased from 406 to 358. While we didn’t observe any shift in agreement over the HRQOL questionnaires between impaired patients and proxies, we did in intact patient with agreement decreasing from fair to poor in Nause and vomiting, Appetite loss and Seizures scales.

The difference in mean scores on the Role functioning scale and the Insomnia scale between impaired patients and proxies became statistically significant while the difference on Social functioning lost statistical significance. On the other hand, while the mean difference between intact patients and their proxies became significant on the Visual disorder and the Itchy skin scale, it lost significance on many scales: Physical Functioning, Cognitive Functioning, Nausea and Vomiting, Pain, Appetite loss, Diarrhoea, Motor dysfunction, Communication deficit, Drowsiness, Hair loss, Weakness in legs and Bladder control.

Altogether we do believe that while the threshold used to distinguish impaired and intact patients can influence the statistical aspects related to differences in mean it does not influence the general level of agreement between patients and proxies and therefore the main outcome of this study. It might be the case that the recurrence of the disease led to a particularly cognitive impaired group but since recurrence is not uncommon in HGG patients we believe our results are not biased.

| Impaired | Mean Proxy 1.5 SD | Mean Proxy  2.0 SD | Mean Patients  1.5 SD | Mean Patients  2.0 SD | CCC 1.5 SD  (C.I) | CCC 2.0 SD  (C.I) | Wilcoxon  p-value 1.5 SD | Wilcoxon  p-value 2.0 SD |
| --- | --- | --- | --- | --- | --- | --- | --- | --- |
| QLQ-C30 † |  |  |  |  |  |  |  |  |
| Global Health | 60.48 | 59.82 | 60.70 | 60.42 | .50 (.42 - .57) | .50 (.42 - .57) | 0.89 | 0.78 |
| Physical Functioning | 71.40 | 73.85 | 74.35 | 70.37 | .73 (.68 - .77) | 0.72 (.67 – .77) | 0.001 | 0.001 |
| Role Functioning | 58.81 | 60.54 | 61.28 | 57.62 | .63 (.52 - .68) | .6 (.53 – .66) | 0.06 | 0.035 |
| Emotional Functioning | 60.03 | 66.76 | 67.06 | 59.84 | .60 (.50 - .68) | .59 (.52 – .65) | 0.001 | 0.001 |
| Cognitive Functioning | 59.58 | 58.05 | 63.90 | 62.8 | .59 (.52 - .65) | 0.58 (.51 – .64) | 0.002 | 0.001 |
| Social Functioning | 62.26 | 61.76 | 64.97 | 64.95 | .56 (.49 - .63) | .55 (.48 – .62) | 0.13 | 0.06 |
| Fatigue | 43.92 | 44.44 | 37.90 | 38.2 | .60 (.52 - .67) | .60 (.53 – .66) | 0.001 | 0.001 |
| Nausea and Vomiting | 3.54 | 3.51 | 4.86 | 4.87 | .44 (.36 - .51) | .45 (.36 – .53) | 0.01 | 0.015 |
| Pain | 17.65 | 18.1 | 15.89 | 16.15 | .58 (.52 - .65) | .58 (.50 – .64) | 0.09 | 0.09 |
| Dyspnoea | 13.02 | 13.33 | 13.33 | 13.33 | .48 (.40 - .55) | .50 (.42 – .58) | 0.74 | 0.95 |
| Insomnia | 29.05 | 28.7 | 26.12 | 25.3 | .59 (.53 - .65) | .61 (.54 – .67) | 0.06 | 0.02 |
| Appetite loss | 9.08 | 9.51 | 10.81 | 11.58 | .50 (.43 - .57) | .52 (.44 – .59) | 0.11 | 0.07 |
| Constipation | 12.35 | 12.55 | 12.13 | 11.86 | .67 (.61 - .72) | .64 (.58 – .70) | 0.75 | 0.47 |
| Diarrhoea | 6.77 | 7.38 | 4.80 | 4.96 | .38 (.29 - .46) | .40 (.32 – 048) | 0.032 | 0.02 |
| Financial difficulties | 20.25 | 20.1 | 19.25 | 18.86 | .51 (.44 - .58) | .50 (.43 – .58) | 0.87 | 0.54 |
| QLQ BN-20 † |  |  |  |  |  |  |  |  |
| Future uncertainty | 45.71 | 45.9 | 43.85 | 43.98 | .58 (.51 - .64) | .58 (.50 – .64) | 0.19 | 0.25 |
| Visual disorders | 16.68 | 17.76 | 17.65 | 18.44 | .67 (.61 - .72) | .66 (.60 – .72) | 0.35 | 0.47 |
| Motor dysfunction | 26.42 | 27.1 | 22.96 | 23.11 | .69 (.63 - .74) | .67 (.61 – .72) | 0.001 | 0.001 |
| Communication deficit | 30.88 | 31.74 | 27.63 | 28.66 | .71 (.66 - .76) | .70 (.65 – .75) | 0.002 | 0.007 |
| Headache | 20.65 | 21 | 20.89 | 21.58 | .67 (.61 - .72) | .66 (.6 – .71) | 0.64 | 0.46 |
| Seizures | 5.12 | 5.7 | 6.03 | 6.07 | .52 (.45 - .59) | .55 (.47 – .62) | 0.46 | 0.78 |
| Drowsiness | 32.16 | 32.86 | 28.35 | 28.76 | .48 (.40 - .522) | .48 (.39 – .55) | 0.004 | 0.009 |
| Hair loss | 11.37 | 10.91 | 10.13 | 10.24 | .54 (.47 - .61) | .56 (.48 – .63) | 0.31 | 0.50 |
| Itchy skin | 8.40 | 8.84 | 10.23 | 10.47 | .45 (.31 - .53) | .46 (.38 – .54) | 0.12 | 0.17 |
| Weakness in legs | 18.94 | 19.65 | 15.79 | 15.9 | .50 (.42 - .57) | .50 (.42 – 0.57) | 0.016 | 0.011 |
| Bladder control | 10.16 | 10.6 | 12.79 | 12.99 | .67 (.61 - .72) | 0.66 (.60 – .72) | 0.01 | 0.026 |

Table 1 Comparison of mean scores, Lin’s CCC and Wilcoxon signed rank coefficient between thresholds for impaired patients

CCC (Concordance Correlation Coefficient); C.I (Confidence interval)

| Intact Patients | Mean Proxy 1.5 SD | Mean Proxy  2.0 SD | Mean Patients  1.5 SD | Mean Patients  2.0 SD | CCC 1.5 SD  (C.I) | CCC 2.0 SD  (C.I) | Wilcoxon  p-value 1.5 SD | Wilcoxon  p-value 2.0 SD |
| --- | --- | --- | --- | --- | --- | --- | --- | --- |
| QLQ-C30 † |  |  |  |  |  |  |  |  |
| Global Health | 70.97 | 69.05 | 71.68 | 68.45 | .5 (.42 - .57) | .43 (.28 – .55) | 0.89 | 0.56 |
| Physical Functioning | 84.82 | 82.8 | 87.64 | 84.27 | .73 (.68 - .77) | .77 (.69 – .83) | 0.001 | 0.26 |
| Role Functioning | 75.81 | 72.93 | 75.71 | 71.99 | .63 (.52 - .68) | .72 (.63 - .79) | 0.06 | 0.64 |
| Emotional Functioning | 69.66 | 66.66 | 72.61 | 71.01 | .60 (.50 - .68) | .63 (.52 – .72) | 0.001 | 0.003 |
| Cognitive Functioning | 78.67 | 76.31 | 80.85 | 77.73 | .59 (.52 - .65) | .58 (.46 – .68) | 0.002 | 0.46 |
| Social Functioning | 79.35 | 76.64 | 79.96 | 73.81 | .56 (.49 - .63) | .61 (.50 – .70) | 0.13 | 0.52 |
| Fatigue | 31.40 | 34.3 | 29.69 | 31.97 | .60 (.52 - .67) | .63 (.52 – .72) | 0.001 | 0.13 |
| Nausea and Vomiting | 2.90 | 3.21 | 3.37 | 3.92 | .44 (.36 - .51) | .26 (.1 – .4) | 0.01 | 0.26 |
| Pain | 12.77 | 13.26 | 11.5 | 12.73 | .58 (.52 - .65) | .66 (.56 – .74) | 0.09 | 0.18 |
| Dyspnoea | 6.23 | 7.3 | 7.45 | 9.49 | .48 (.40 - .55) | .42 (.28 – .54) | 0.74 | 0.18 |
| Insomnia | 27.11 | 28.3 | 27.66 | 29.74 | .59 (.53 - .65) | .62 (.50 – .70) | 0.06 | 0.50 |
| Appetite loss | 6.09 | 6.19 | 5.73 | 5.71 | .5 (.43 - .57) | .3 (14 - .44) | 0.11 | 0.86 |
| Constipation | 12.45 | 11.94 | 14.18 | 15.17 | .67 (.61 - .72) | .74 (.66 – .81) | 0.75 | 0.15 |
| Diarrhoea | 3.41 | 3.03 | 3.94 | 3.79 | .38 (.29 - .46) | .35 (.2 – .48) | 0.03 | 0.63 |
| Financial difficulties | 19.57 | 19.7 | 10.64 | 15.33 | .51 (.44 - .58) | .38 (.24 – .51) | 0.87 | 0.38 |
| QLQ BN-20 † |  |  |  |  |  |  |  |  |
| Future uncertainty | 38.65 | 40.33 | 36.35 | 38.32 | .58 (.51 - .64) | .6 (.48 – .69) | 0.19 | 0.37 |
| Visual disorder | 8.18 | 7.84 | 10.75 | 10.46 | .67 (.61 - .72) | .64 (.54 – .73) | 0.35 | 0.019 |
| Motor dysfunction | 11.23 | 14.33 | 11.83 | 14.53 | .69 (.63 - .74) | .79 (.72 – .84) | 0.001 | 0.86 |
| Communication deficit | 9.22 | 13.87 | 11.59 | 13.95 | .71 (.66 - .76) | .69 (.6 – .77) | 0.002 | 0.99 |
| Headache | 17.58 | 16.91 | 16.13 | 16.16 | .67 (.61 - .72) | .7 (.6 – .78) | 0.64 | 0.64 |
| Seizures | 3.19 | 2.47 | 4.4 | 4.7 | .52 (.45 - .59) | .29 (.15 – .42) | 0.46 | 0.08 |
| Drowsiness | 20.07 | 22.63 | 18.48 | 19.90 | .48 (.40 - .522) | .54 (.41 – .65) | 0.004 | 0.19 |
| Hair loss | 8.70 | 8.65 | 7.53 | 7.12 | .54 (.47 - .61) | .56 (.43 – .67) | 0.31 | 0.32 |
| Itchy skin | 5.13 | 5.26 | 12.32 | 10.52 | .45 (.31 - .53) | .43 (.29 – .54) | 0.12 | 0.005 |
| Weakness in legs | 8.24 | 9.88 | 9.06 | 10.86 | .50 (.42 - .57) | .59 (.47 – .69) | 0.016 | 0.54 |
| Bladder control | 6.38 | 6.47 | 6.81 | 7.91 | .67 (.61 - .72) | .76 (.69 – .82) | 0.01 | 0.18 |

Table 2

Table 21 Comparison of mean scores, Lin’s CCC and Wilcoxon signed rank coefficient between thresholds for intact patients

CCC (Concordance Correlation Coefficient); C.I (Confidence interval)

**Comparative analysis for excluded patients**

The original sample consisted of 731 patients and 691 proxies. Patients were included based on different criteria:

*Histological criteria*

To be included, patients had to have a histological diagnosis of either Glioblastoma, Giant Cell Glioblastoma, Gliosarcoma, Glioblastoma with an oligodendroglial component, or Astrocytoma WHO grade III. This led to the exclusion of 103 patients that did not meet histological criteria.

| \| *Excluded histologies:* \| \| \| \| \| \| \| \| --- \| --- \| --- \| --- \| --- \| --- \| --- \| \|  \| Frequency \| \| Percent \| \| \| astrocytoma WHO grade II \| \| 50 \| \| 48,5 \| \| \| oligoastrocytoma WHO grade II \| \| 34 \| \| 33,0 \| \| \| oligodendroglioma WHO grade I \| \| 19 \| \| 18,4 \| \| \| Total \| \| 103 \| \| 100,0 \| \|   *Included histologies:* | | | | | | |
| --- | --- | --- | --- | --- | --- | --- | --- | --- | --- | --- | --- | --- | --- | --- | --- | --- | --- | --- | --- | --- | --- | --- | --- | --- | --- | --- | --- | --- | --- | --- | --- | --- | --- | --- | --- | --- | --- | --- | --- | --- | --- | --- |
|  | Frequency | | Percent | |  |  |
| Glioblastoma | | 105 | | 82,0 | |  |
| Giant Cell Glioblastoma | | 4 | | 3,1 | |  |
| Glioblastoma with an oligodenroglial component | | 6 | | 4,7 | |  |
| astrocytoma WHO grade III | | 13 | | 10,2 | |  |
| Total | | 128 | | 100,0 | |  |

*HRQOL*

Out of the 128 patients that met the histological criteria, 36 patients had missing HRQOL evaluations, 36 patients had no proxy HRQOL evaluations, and 16 patients had HRQOL and NCF evaluations that were more than ± 7 days apart from each other.

These criteria led to the exclusion of 88 patients.

*NCF*

40 patients had incomplete NCF evaluations with at least one missing test outcome:

| *TEST OUTCOMES MISSING* | | | |
| --- | --- | --- | --- |
|  | Frequency | Percent |  |
| 1 Test outcome | 15 | 37,5 |  |
| 2 Test outcome | 13 | 32,5 |  |
| 3 Test outcomes | 4 | 10,0 |  |
| 4 Test outcomes | 1 | 2,5 |  |
| 5 Test outcomes | 4 | 10,0 |  |
| 6 Test outcomes | 3 | 7,5 |  |
| Total | 40 | 100,0 |  |

| **Descriptive Statistics of the neurocognitively impaired** | | | | | |
| --- | --- | --- | --- | --- | --- |
|  | N | Minimum | Maximum | Mean | Std. Deviation |
| Total Recall | 34 | -6,40 | ,15 | -3,3631 | 1,88377 |
| Delayed Recall | 31 | -5,44 | ,46 | -3,4942 | 1,98427 |
| Delayed Recognition | 29 | -9,14 | ,93 | -2,5146 | 2,81442 |
| COWA | 33 | -4,03 | -,23 | -2,3928 | 1,21428 |
| TMT_A | 16 | -5,39 | 2,08 | -1,9320 | 2,43781 |
| TMT_B | 2 | -1,88 | -1,77 | -1,8251 | ,08236 |

An independent samples t-test was conducted to compare the test scores between the included (impaired) and excluded groups. Due to the limited sample size of the participants with available TMT-B test outcomes, a meaningful t-test for this specific outcome measure could not be performed. The mean scores for the remaining five test outcomes of the included group were compared to the mean scores of the excluded group. We found significant differences between the two groups on the Hopkins verbal learning test - Revised outcome measures Total Recall, Delayed Recall and on the COWA.

Hopkins Total Recall mean scores of the included group (M = -2.35, SD = 1.44) were compared to the mean scores of the excluded group (M = -3.36, SD = 1.88).

Levene's Test for equality of variances indicated that the variances were not equal, F (1, 438) = 7.039, *p* = 0.008. The two-sided t-test with equal variances not assumed showed a significant difference between the groups, *t* (36.347) = 3.072, *p* = 0.004. The effect size was medium, Cohen's d = 0.689. These results indicate that the group of patients included in the study performed significantly better on the Hopkins Total Recall test compared to the group excluded from the study.

The Hopkins Delayed Recall mean scores of the included group (M = -2.7, SD = 1.71) were compared to the mean scores for the excluded group (M = -3,49, SD = 1,98).

Levene's Test for Equality of Variances indicated that the variances were equal, F (1, 435) = 2.192, *p* = 0.139. The two-sided t-test with equal variances assumed showed a significant difference between the groups, t (435) = 2.43, p = 0.015. The effect size was small, Cohen's d = 0.453. These results suggest that the group of patients included in the study performed significantly better on the Hopkins Delayed Recall test compared to the group excluded from the study.

The COWA mean scores of the included group (M = -1.88, SD = 1.17) were compared to the mean scores for the excluded group (M = 2,39, SD = 1.21).

Levene's Test for Equality of Variances indicated that the variances were equal, F (1, 437) = 1.514, p = 0.219. The two-sided t-test with equal variances assumed showed a significant difference between the groups, *t* (437) = 2.37, *p* = .018. The effect size was small, Cohen's *d* = 0.429. These results also suggest that the group of patients included in the study performed significantly better on the COWA test compared to the group excluded from the study.

Owing to the considerable difference in sample size of included and excluded patients it is hard to draw definitive conclusions from these comparisons. Furthermore, defining a cutoff pertaining to an acceptable number of missing test outcomes to allow a patient to be included in the study is more or less arbitrary.
